# Symptom burden and relief in palliative care units of German Comprehensive Cancer Center and other hospitals

**DOI:** 10.1007/s00432-023-05557-6

**Published:** 2024-03-27

**Authors:** Julia Berendt, Sarah Brunner, Maria Heckel, Mitra Tewes, Christoph Ostgathe, Susanne Gahr

**Affiliations:** 1 Department of Palliative Medicine and Comprehensive Cancer Center, CCC Erlangen-EMN, University Hospital Erlangen Friedrich-Alexander-Universität Erlangen-Nürnberg, Krankenhausstraße 12, 91054 Erlangen, Germany; 2 Medical Informatics and Communication Center and Comprehensive Cancer Center, CCC Erlangen-EMN, University Hospital Erlangen Friedrich-Alexander-Universität Erlangen-Nürnberg, Erlangen, Germany; 3 Department of Palliative Medicine, West German Cancer Center, University Hospital Essen, University of Duisburg-Essen, Essen, Germany

**Keywords:** Symptom burden, Symptom relief, Palliative care, Registry, Cancer

## Abstract

**Purpose:**

The National Hospice and Palliative Registry contains patient data from German hospice and palliative care facilities about symptoms. The aim of the study at hand is to differentiate symptom burden of patients in palliative care units between Comprehensive Cancer Center (CCC) and other hospitals regarding symptom burden and relief of patients in palliative care units.

**Methods:**

The registry analysis provided data of patients in palliative care units (2014–2018). We analyzed characteristic and symptom-related data on 18 symptoms, with considerable symptom-burdened patients (moderate or severe). We followed a cancer (yes/no) and facility-specific descriptive analysis (*f*, %, *μ*, Mdn, SD, *V*, *r*) using SPSS.

**Results:**

We evaluated 10,447 patient records (CCC: 4234 pts/non CCC 6,213 pts), 82% with a cancer diagnosis. For cancer patients, the mean age in CCC-affiliated palliative care units was 68 (SD 19–99) years, in others 73 (SD 23–104) years (*p* < 0.05; *V* = 0.2). The proportion of patients with significant symptom burden is lower in CCC-affiliated than in other palliative care units. The difference between facilities shows a significant weak effect in pain, vomiting and constipation, depressiveness, anxiety, and tension. The proportion of cases which symptom burden could be alleviated is higher in CCC-affiliated palliative care units with significant weak/medium effect in pain, nausea, vomiting, shortness of breath, constipation, wound care problems, depressiveness, anxiety, tension, confusion, and problems in organizing care.

**Conclusion:**

We found differences in symptom burden and symptom relief between CCC-affiliated and other palliative care units. CCCs should continue to feel responsible for sharing knowledge about symptom relief, such as through standard operating procedures and education.

## Introduction

In 2009, the German Cancer Aid founded the Comprehensive Cancer Center (CCC) Network: an alliance of oncological centers of excellence. Oncological centers offer patients and their families optimal, individualized health care based on the most recent evidence (Albreht et al. 2017). CCCs are high-performance medical centers, especially for modern cancer therapy, located in high population density areas and highly specialized in treating complex and difficult cases. Those certified centers declare a survival benefit of treatment (Schoffer et al. 2022). CCCs develop a role model effect for patient-centered cancer care of stakeholders in cancer care (Brandts 2019). Regarding the interest of interdisciplinary care palliative care facilities are affiliated with a CCC (Berendt et al. 2016). The Palliative Care Guideline from the National Cancer Center Network (NCCN) reports that high symptom burden may require palliative care consultation (Dans et al. 2022). The World Health Organization defines palliative care as “an approach that improves the quality of life of patients and their families facing the problems associated with life-threatening illness. It prevents and relieves suffering through the early identification, correct assessment and treatment of pain and other problems […]. Palliative care is required for a wide range of diseases […]” (WHO 2020). The concept of palliative care explicitly contains the relief of physical, psychological, social, and other problems (Mori et al. 2011; Radbruch et al. 2020). For high-quality standards in cancer care, CCC develop clinical practice guidelines for health care professionals and keep them up to date, especially to manage symptoms and problems according to current research findings (Berendt et al. 2017; Lödel et al. 2020). Symptom relief is highly successful at all settings in palliative care (Delgado-Guay et al. 2018; Jung et al. 2022; Ullrich et al. 2017). CCC are usually integrated within an university hospital. Therefore, palliative care is also provided to patients without cancer. As patients differ substantially (Stiel et al. 2014; Ostgathe et al. 2011), it is always important to consider both groups, on the one hand to better understand the specificity of needs of palliative care patients with cancer, to better taylor services and to mutually learn in regard, e.g., to symptom assessment and management.

In 2009, the German Association for Palliative Medicine (DGP) and the German Hospice and Palliative Association (DHPV) established the German National Hospice and Palliative Registry. It based on the Edmonton Symptom Assessment Scale (ESAS) (Hui and Bruera 2017; Stiel et al. 2010) and underwent some revisions after validation (Stiel et al. 2012). The title of the validated data-collecting instrument was Hospice and Palliative Care Evaluation Symptom and Problem Checklist (HOPE-SP-CL). The documentation contains a symptom and problem checklist on physical, psychological, social, and care-related symptoms (Stiel et al. 2012), and is noted essential in the Best Practice Recommendations for Integrating Palliative Care into CCCs funded by the German Cancer Aid (Berendt et al. 2016). Documented information came from palliative care teams, inpatient hospices, hospital palliative care support teams, and palliative care units. The current literature presents limited evidence on institution-specific analysis.

The aim of this study is to report (1) the symptom burden of patients on admission to palliative care units of CCCs and other hospitals, and (2) the success in symptom relief between admission and the end of treatment in each institution type. According to our hypothesis, palliative care units of CCCs treat patients with more considerable symptoms than other hospitals.

## Methods

### Register data

Our reporting is based on RECORD items for “Routinely collected health Data” as we use data from the German National Hospice and Palliative Registry. In February 2020, anonymized data (2014–2018) were provided by Smart-Q GmbH that collects and manages registry data of 184 institutions. With the given data set, it was only possible to determine whether the data came from the palliative care unit or the palliative care consultation service of a hospital. Eleven German CCCs agreed to declare the datasets back to the institution type “CCC”. Another reference to the institution "hospital" does not exist. The data set for the consultation services was eliminated due to missing data. We labeled the source of the remaining data sets as "palliative care unit of other hospitals", if they were obtained from palliative care units, e.g., of other university hospitals not certified as CCC.

Besides demographic, disease-specific, and treatment-related information, the core data set of the registry contains the validated symptom and problem checklist HOPE-SP-CL (Stiel et al. 2012). The HOPE-SP-CL encompasses proxy assessed data on pain, nausea, vomiting, dyspnea, constipation, weakness, loss of appetite, tiredness, wound care problems, need of assistance with activities of daily living, feeling depressed, anxiety, tension, restlessness, disorientation/confusion, problems with organization of care, overburdening of family, and other problems. These symptoms’ intensities are ranked on a scale from "0 = none", "1 = mild", "2 = moderate", to "3 = severe" and were assessed at two time points: at (1) admission, and (2) at the end of the treatment (hospital discharge or death).

### Sample

This study analyzed data sets of patients hospitalized in German palliative care units between 2014 and 2018 entered in the register by the institution providing palliative care.

### Transformation of scale

We declared all patients who rated their symptoms with 2 (moderate) or 3 (severe) as considerably burdened. The change of symptom burden was measured by the numerical difference between symptom burden at the beginning and end of treatment. In the registry, the end of treatment is defined as last information before discharge or death. Neutral values referred to a constant state. If there were positive values (symptom intensity increased), a decrease in symptom intensity was indicated. Negative values (symptom intensity decreased) showed an improvement, designated as symptom burden relief.

### Study design

The study design is quantitative and descriptive, using exploratory data analysis (cohort study). A correlation analysis (rank correlation) between two variables is conducted: institution type (independent variable) and each symptom (dependent variable).

### Analysis

**Fig. 1:**
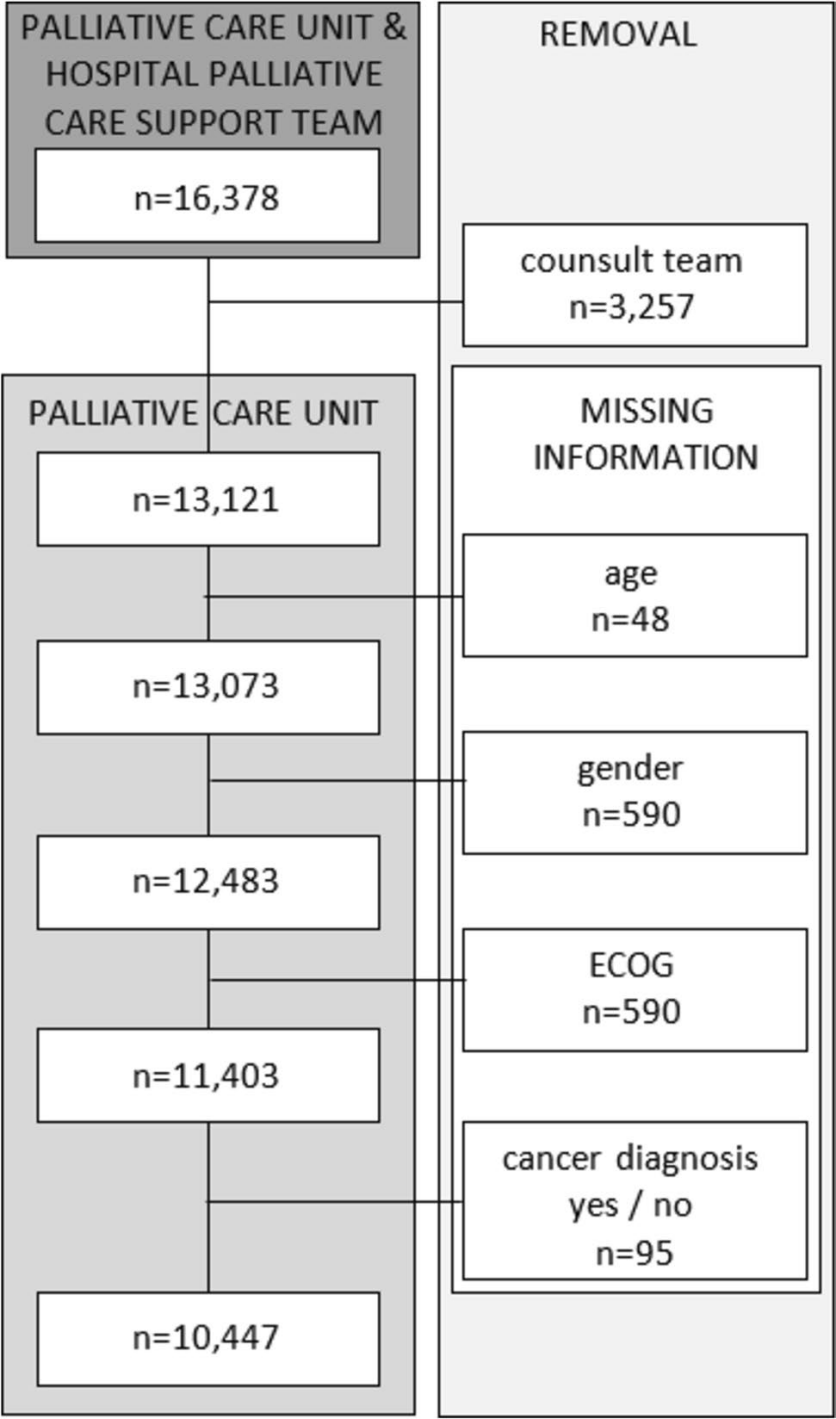
Data cleaning; removal of cases with missing data in different categories

The data set was adjusted at baseline for missing information regarding age, sex, cancer diagnosis, and ECOG (Eastern Cooperative Oncology Group) Performance Status (see Fig. 1).

Data were collected using IBM SPSS Statistics 24. The frequencies (*n*), percent (%), mean (*M*), standard deviation (SD), median (mdn), significance (*p*), Cramér’s *V* (*V*), and Pearson's *r* (*r*) were calculated. The level of significance was set at 5%. Because of a large sample size, Chi^2^ tests and Chi^2^-based measures such as Cramér’s *V* according to Cohen (Cohen 1988) and Pearson’s correlations *r* were performed to derive insights for practical relevance. Cramér’s *V* is based on the contingency coefficient phi *φ*. We differentiated between two evaluation streams for analysis of patient clientele and symptom relief: cancer-directed (cancer and non-cancer), and institution-specific (CCC-affiliated palliative care units vs. other palliative care units). Percent differences were considered above a 5% deviation and were analyzed in relation to effect size.

## Results

### Data set

We analyzed 10,447 patient records: 4234 cases of CCC-affiliated palliative care units (41%) and 6213 cases of other palliative care units (59%). The total data set included 8600 cases with cancer (82%) and 1847 patients with a non-cancer diagnosis (18%) (Table 1).

**Table 1:** Distribution of primary diagnosis

Primary diagnosis	ICD-10	*n*	%
Non-cancer diagnosis		1847	17.7
Malignant neoplasms of lip, oral cavity and pharynx	C00–C14	265	2.5
Malignant neoplasms of digestive organs	C15–C26	2394	22.9
Malignant neoplasms of respiratory and intrathoracic organs	C30–C39	1948	18.6
Malignant neoplasms of bone and articular cartilage	C40–C41	36	0.3
Malignant melanoma of skin	C43–C44	197	1.9
Malignant neoplasms of mesothelial and soft tissue	C45–C49	188	1.8
Malignant neoplasm of breast	C50	677	6.5
Malignant neoplasms of female genital organs	C51–C58	537	5.1
Malignant neoplasms of male genital organs	C60–C63	495	4.7
Malignant neoplasms of urinary tract	C64–C68	498	4.8
Malignant neoplasms of eye, brain and other parts of central nervous system	C69–C72	317	3.0
Malignant neoplasms of thyroid and other endocrine glands	C73–C75	57	0.5
Malignant neoplasms of ill-defined, secondary and unspecified sites	C76–C80	558	5.3
Malignant neoplasms, stated or presumed to be primary, of lymphoid, hematopoietic and related tissue	C81–C96	433	4.1
Overall		10,447	100

### Characteristics of cancer patients

Among cancer patients in CCC-affiliated palliative care units, 46.0% are female, compared with 48.1% in other palliative care units. There is no correlation between institution type and gender or ECOG. Cancer patients in a CCC-affiliated palliative care unit are five years younger within a detectable weak effect and have fewer symptoms at the time of admission. Cancer patients remained hospitalized in CCC-affiliated palliative care unit one day shorter on median than in other palliative care units, at a moderate effect. There was a higher proportion of cancer patients dying in the palliative care unit of CCCs than other hospitals showing a weak significant effect. People not diagnosed with cancer but receiving treatment in a CCC are also younger, but have a shorter duration of stay (Table 2).

**Table 2:** Characteristics at admission of palliative care unit for each type of institution (*n* = 10,447)

	Cancer *n* = 8600				Non-cancer (*n* = 1847)			
	CCC-affiliated palliative care units n = 3314	Other palliative care units *n* = 5286	effect size	Significance	CCC-affiliated palliative care units *n* = 920	Other palliative care units *n* = 927	effect size	Significance
			Cramér’s *V*	*p* value			Cramér’s *V*	*p* value
Age Ø (in years)	**68 [19–99]**	**73 [23–104]**	**0.23**	**< 0.05**	**74 [20–100]**	**80 [20–105]**	**0.33**	**< 0.05**
Gender, female (%)	46.0	48.1	0.02	0.057	47.6	51.3	0.04	
ECOG^b^ (average)	3.2	3.0	0.09	< 0.05	3.6	3.5	0.08	< 0.05
ECOG^b^ (median)	3	3	0.09	< 0.05	4	4	0.08	< 0.05
Duration of stay (median)	**9**	**10**	**0.14**	**< 0.05**	**8**	**8**	**0.25**	**< 0.05**
Number of symptoms^a^ (median)	**10**	**12**	**0.32**	**< 0.05**	**8**	**11**	**0.38**	**< 0.05**
Living situation (%)			0.07	< 0.05			**0.10**	**< 0.05**
Alone	18.0	23.4			18.3	17.0		
Nursing home	8.9	7.3			13.3	22.7		
Relatives/reference person	48.9	63.9			37.6	54.3		
Other	0.9	1.3			1.3	1.8		
No information	23.2	4.1			29.6	4.3		
End of treatment (%)			**0.11**	**< 0.05**			**0.12**	**< 0.05**
Deceased	49.5	42.1			46.2	52.1		
Transfer/discharge	47.5	56.3			48.5	46.1		
Stabilization…	–	–			0.2	–		
Other	1.1	0.2			–	0.1		
No information	1.9	1.4			2.7	1.7		
Place of death (%)			**0.15**	**< 0.05**			**0.12**	**< 0.05**
At home	0.2	1.0			-	0.2		
Asylum	0.1	0.1			0.2	0.1		
Hospice	0.6	0.8			0.4	0.2		
Palliative care unit	44.5	38.0			44.6	47.5		
Hospital	7.6	3.2			7.1	3.7		
Other	–	–			–	–		
No information	46.9	56.9			**48.0**	**48.3**		

Bold implies a significant correlation, effect size Cramér’s *V*: 0 = negligible/0.10 = small/0.3 = medium/0.50 = large

^a^documented symptoms with symptom burden 1 = mild/2 = moderate/3 = severe, *mdn* median

^b^*ECOG* Eastern Cooperative Oncology Group functional status: 0 = fully active/1 = moderately limited physical activity, able to work/, 2 = disabled, increasing need for care/3 = mostly external supply; confined to bed or chair ˃ 50%/4 = completely disabled; totally confined to bed or chair

### Symptom burden for cancer patients according to institution type

The rate of considerably symptom-burdened patients is more than 10% higher for each symptom in other palliative care units than in CCC-affiliated palliative care units. Thus, in CCC-affiliated palliative care units, significantly fewer cancer patients are affected by pain, vomiting and constipation, depressiveness, anxiety, tension at a weak effect between institution types (Table 3).

**Table 3:** Percentage of considerable symptom-burdened patients^a^ at admission of palliative care unit, differentiated according to cancer or non-cancer and type of institution (*n* = 10,447)

Considerable symptom-burdened patients^*b*^	Cancer *n* = 8600				Non-cancer *n* = 1847			
	CCC-affiliated palliative care units	Other palliative care units	Effect size	Significance	CCC-affiliated palliative care units	Other palliative care units	Effect size	Significance
	%	%	Cramér’s *V*	*p* value	%	%	Cramér’s *V*	*p* value
Pain	**49.8**	**62.4**	**0.12**	**< 0.05**	**29.6**	**45.8**	**0.17**	**< 0.05**
Nausea	22.2	29.9	0.08	< 0.05	10.1	14.4	0.07	< 0.05
Vomiting	**11.2**	**22.0**	**0.13**	<** 0.05**	**4.9**	9.5	0.09	< 0.05
Dyspnea	32.3	39.1	0.07	< 0.05	**33.8**	**47.7**	**0.14**	<** 0.05**
Constipation	**34.6**	**44.4**	**0.10**	<** 0.05**	**26.1**	**39.1**	**0.13**	<** 0.05**
Weakness	89.8	89.8	0.00	0.984	93.2	92.9	0.01	0.831
Loss of appetite	71.6	73.0	0.01	0.210	71.4	72.9	0.02	0.529
Tiredness	65.2	73.0	0.08	< 0.05	66.1	74.7	0.09	< 0.05
Wound care problems	20.2	17.7	0.03	< 0.05	22.2	25.5	0.04	0.149
Need of assistance with activities of daily living	81.3	80.3	0.01	0.310	88.7	90.0	0.02	0.407
Feeling depressed	**29.2**	**38.7**	**0.10**	<** 0.05**	27.3	27.6	0.00	0.887
Anxiety	**33.0**	**47.3**	**0.14**	<** 0.05**	31.7	39.4	0.08	< 0.05
Tension	**40.0**	**51.6**	**0.11**	<** 0.05**	**35.7**	**45.7**	**0.10**	<** 0.05**
Restlessness	0.1	0	0.02	0.112	1.2	0.3	0.05	0.081
Disorientation/confusion	17.3	20.4	0.04	< 0.05	34.4	36.1	0.02	0.507
Problems with the organization of supply	73.1	68.4	0.05	< 0.05	**73.8**	**63.3**	**0.11**	<** 0.05**
Overburdening of family	75.8	75.2	0.01	0.610	74.6	68.6	0.07	< 0.05
Other problems	55.1	52.4	0.03	0.395	**75.8**	**50.0**	**0.26**	<** 0.05**

Bold implies a significant correlation, effect size Cramér’s *V*: 0 = negligible/0.10 = small/0.3 = medium/0.50 = large

^a^Symptom burden/intensity “2 = moderate" to "3 = severe”

### Symptom burden relief for cancer patients according to institution type

In CCC-affiliated palliative care units, symptom burden reduced to a higher rate among significantly symptom-burdened cancer patients compared with other palliative care units, especially with significant weak or moderate effect in pain, nausea, vomiting, dyspnea, constipation, wound care problems, feeling depressed, anxiety, tension, disorientation/confusion, and problems with organization of care (Table 4).

**Table 4:** Percentage of considerable symptom-burdened patients^a^ with achieved symptom relief of palliative care units, differentiated according to cancer or non-cancer and type of institution (*n* = 10,447)

Considerable-symptom-burdened patients^b^	Cancer *n* = 8600				Non-cancer *n* = 1847			
	CCC-affiliated palliative care units	Other palliative care units	Effect size	Significance	CCC-affiliated palliative care units	Other palliative care units	Effect size	Significance
	%	%	Cramér’s *V*	*p* value	%	%	Cramér’s *V*	*p* value
Pain	**74.9**	**48.4**	**0.25**	<** 0.05**	**74.2**	**50.9**	**0.22**	<** 0.05**
Nausea	**79.3**	**50.9**	**0.26**	<** 0.05**	**77.8**	**50.5**	**0.27**	<** 0.05**
Vomiting	**79.7**	**41.6**	**0.31**	<** 0.05**	**80.6**	**40.3**	**0.37**	<** 0.05**
Dyspnea	**63.1**	**40.4**	**0.21**	<** 0.05**	54.9	45.4	0.09	< 0.05
Constipation	**49.9**	**36.2**	**0.13**	<** 0.05**	**52.3**	**40.7**	**0.11**	<** 0.05**
Weakness	19.7	18.8	0.01	0.347	13.9	17.5	0.05	0.053
Loss of appetite	28.9	25.5	0.04	< 0.05	23.0	23.8	0.01	0.764
Tiredness	25.8	22.7	0.03	< 0.05	20.6	23.1	0.03	0.348
Wound care problems	**42.9**	**32.7**	**0.10**	<** 0.05**	38.7	35.0	0.04	0.517
Need of assistance with activities of daily living	17.5	16.0	0.02	0.140	10.7	12.6	0.03	0.312
Feeling depressed	**55.7**	**27.4**	**0.26**	<** 0.05**	**43.8**	**29.9**	**0.14**	<** 0.05**
Anxiety	**58.3**	**32.9**	**0.23**	<** 0.05**	**52.3**	**37.6**	**0.14**	<** 0.05**
Tension	**58.5**	**32.3**	**0.24**	<** 0.05**	**54.5**	**39.3**	**0.14**	<** 0.05**
Restlessness	**0**	**100**	**1.00**	**0.083**	**0**	**100**	**1.00**	<** 0.05**
Disorientation/confusion	**36.0**	**25.3**	**0.11**	<** 0.05**	31.9	28.1	0.04	0.406
Problems with the organization of supply	**51.1**	**35.4**	**0.15**	<** 0.05**	43.0	39.5	0.04	0.303
Overburdening of family	40.3	30.9	0.09	< 0.05	32.1	35.6	0.04	0.276
Other problems	36.1	43.7	0.08	0.154	**17.0**	**44.0**	**0.29**	<** 0.05**

Bold implies a significant correlation, effect size Cramér’s *V*: 0 = negligible/0.10 = small/0.3 = medium/0.50 = large

^a^Symptom burden/intensity “2 = moderate" to "3 = severe”

## Discussion

The study at hand is the first using nationwide registry data for cross-institutional analysis regarding symptom burden and relief in palliative care of German CCC and other hospitals. Interestingly and at the first sight somewhat counterintuitive, we found a larger proportion of considerably symptom-burdened cancer patients being treated in palliative care units of other hospitals compared to CCC-affiliated palliative care units for a range of symptoms. A result contrasting with our hypothesis and previous findings (Brunner et al. 2022; Delgado-Guay et al. 2018). However, Brunner et al. (2022) considered all patients over all classifications for symptom intensity, whereas we focused on cases with moderate and severe symptom burden.

CCC-affiliated palliative care units treat patients younger on average of five years. The lower proportion of considerably symptom-burdened cancer patients may be explained for CCC-affiliated palliative care units by younger patients with similar variance. One reason is the generally young patient clientele in CCCs. Internationally, the average age of cancer patients and in CCCs is low regarding 59–68 years (Bryson et al. 2010; Jung et al. 2022; Hui et al. 2010). Urban centers and metropolises imply a young demographic population. For another reason, comorbidities that may impact symptom burden occur primarily at an older age (Canoui-Poitrine et al. 2022; Williams et al. 2016).The interpretation of the data is challenging. The observation that in CCCs the number of considerably burdened patients is lower may also be an effect of timely integration of specialized palliative care alongside cancer treatment on the oncological wards positively impacting on the quality of symptom management. On the other hand, we see more patients dying in CCC palliative care units, even with a somewhat younger age structure. As CCCs apply novel cancer therapies and research-based procedures patients may often referred to inpatient specialized palliative care at a rather advanced stage of their disease (Hui et al. 2017). As a result, more cancer patients may die in the palliative care unit of a CCC despite a younger age structure. Palliative care units present very severe, complex cases as shown according to ECOG status in both: CCC-affiliated palliative care units and other palliative care units (Brunner et al. 2022). Every second cancer patient reports moderate or severe pain in German palliative care units. Internationally, the ratio of patients with moderate and severe pain is lower, on average 38% (32–43%) (van den Beuken-van Everdingen et al. 2016). The guideline "Palliative Care" of the NCCN refers to a high symptom burden, as a decisive factor to consult palliative care (Dans et al. 2022). Pain, depression, anxiety, and tension are increased reported by cancer patients in other palliative care units. That often occur as cluster (Öhlen et al. 2017; Stiel et al. 2014). Depressiveness and anxiety are pronounced, especially when the cancer has been diagnosed (Vogt et al. 2021).

CCC-affiliated palliative care units predominantly improve symptoms in considerably distressed patients. This is consistent with previous findings (Delgado-Guay et al. 2018). The difference between institutions in favor of CCC-affiliated palliative care units was shown for pain, anxiety, lack of appetite, as well as sleep quality (Jung et al. 2022). We show how CCC-affiliated palliative care units also reduce other distressing problems among cancer patients, such as nausea, vomiting, shortness of breath, constipation, wound care problems, depressive symptoms, tension, confusion, and problems in the care setting. CCCs understand their role in treating symptoms according to the latest scientific findings and sharing guidelines, standards, and recommendations. It is important to create, update, disseminate and use guidelines and Standard Operating Procedures for general and specialized palliative care. Relieving physical symptoms is also reflected in the Standard Operating Procedures developed by the Palliative Care Working Group in the CCC Network, where the largest proportion is symptom-based (Lödel et al. 2020; Ostgathe et al. 2017; Stachura et al. 2017). The Standard Operating Procedures are primarily focused on cancer patients because they were developed within the framework of the CCCs. They may also be beneficial for patients who suffer from other diseases than cancer in particular when no other Standard Operating Procedures are available. The use of a Standard Operating Procedure must be critically reflected in each individual case, since the pathophysiology of, e.g., symptoms can be very different and not all strategies can be transferred 1 to 1 to others, e.g., more careful administration of opioids in cardiac insufficiency (Gaertner et al. 2023).

However, not only physical symptoms should be considered in Standard Operating Procedures. Emotional problems such as and tension often occur (van den Beuken-van Everdingen et al. 2016), especially for younger patients (Abdelaal et al. 2023). So the working group members developed Standard Operating Procedures for depression (Schwartz et al. 2022) and anxiety (Hornemann et al. 2022). Psychological symptom burden, in particular anxiety, must be well documented to provide appropriate multiprofessional offers at an early stage. Besides the importance of the documentation of psychosocial symptoms, collaboration with psychooncology as soon as symptoms become apparent could benefit synergies (Zucca et al. 2015).

The findings focus on cancer patients. Nevertheless, the results may serve as a motivation for CCCs and non-CCCs to collaborate more and share knowledge to better taylor support and services for both groups.

Overall, the study shows that palliative inpatient care reduces symptom burden, independent of CCC-affiliated or other palliative care units. Providing palliative care that achieves consistent, all-encompassing symptom relief on a physical, psychological, social, and spiritual level is a way to enhance or maintain the patient's quality of life (Radbruch et al. 2020). All institution types satisfy the requirement of care. Both CCC-affiliated and other palliative care units improve symptom burden to a significant degree, especially in the physical, psychological, and social dimension. This is achieved among cancer patients even in CCC-affiliated palliative care units at a median shorter duration of care. The average length of care is similar in palliative care of NCCN (Brunner et al. 2022; Calton et al. 2016). Thus, palliative care reduces symptom distress effectively (Gomes et al. 2016), cost-effectively (Smith et al. 2014), and consequently regardless of place of care. CCCs are responsible for providing structures, information and training in palliative care to overcome hurdles (Anderson et al. 2022).

Besides the difference in symptom burden between CCC-affiliated and other palliative care units, the differences could also be caused due to other factors, such as the distribution of principal diagnoses. More research is needed to address this. Knowledge of the differences in symptom burden among patients in CCC-affiliated and other palliative care units could be helpful to improve the approach to symptom burden as well as the structures of palliative care for oncology patients in CCCs and other hospitals. It should raise the awareness that symptom burden relief in the context of palliative care makes a significant contribution to maintaining the quality of life of cancer patients.

## Limitation

The Registry does not contain institution-specific information, such as staff, beds or type and hence no comparison in regard to further structural data (e.g., staffing, multiprofessionality, independent or integrated department, hospital size) was possible and, therefore, possible structural confounders may be overseen. The group “other hospitals” includes general hospitals, and also university hospitals, and oncology centers. The data composition affects the interpretation of results. Also a misclassification bias or reporting bias may impact the data. The focus of the analysis is not on the patient’s diagnosis. But, we are clear about cancer diagnoses may differ between the settings as patients with rare cancers are preferably treated in specialized centers. More details are given in another publication (Brunner et al. 2022). The analysis rather pursues an overview of the patient data: physical, psychological, and social care situation. Spiritual aspects are not documented in the registry, although via "other problems". Nevertheless, physical and emotional problems can be well compared. Excessive demand seems to be an indirect indicator for a stressed family.

The patient datasets varies within each CCC (from 13 to 1438) and per documentation year. Technical, financial and human resources influence data quality and transmission of data sets to the registry. Documenter's subjective assessment, level of training, and professional group assignment distort data. Information may not always be complete in visits or admission conversations. Symptoms were assessed by proxies, reducing data quality. However, in many patients on the palliative care units due to cognitive impairment and increasing frailty due to the disease self-assessment is not possible. Proxy assessment is particularly challenging for patients who are nearing death, but also beneficial. First, it is simpler to identify, rank, and monitor goals for problem-solving. Second, it ensures complete data collection and raises awareness of general needs and symptoms (Campbell et al. 2022). For consistent data analysis, we decided to consider only data that was collected completely (both at the beginning and at the end of treatment) and exclude patient registrations with missing data regarding one or the other point in time. We assume that the main symptoms are included. But it is obvious that measurements of symptom burden relief in dying patients may not be comparable to measurements in patients discharged from the unit. This also distorts the results. Aspects of timing for palliative care integration in the palliative care units are not considered. Nonetheless, this analysis is the first multicenter analysis of patient data. Using a large sample, we prove and disprove findings from the literature and validate them with the additional calculated effect size. We are interested to conduct more multicenter studies to identify specifics in the care of critically ill patients among institutions and further improve care across all institution types.

## Conclusion

The role of CCCs in the treatment of special patient collectives (younger cancer patients), timely integration and transferring knowledge to other areas is evident from the result, for example via Standard Operating Procedures. Furthermore, it is important to ensure general and specialized palliative care being systematically integrated in all hospitals and not left to oncological competence centers alone. Moreover, it shows to promote general palliative care in hospital institutions to better address physical and emotional symptoms and problems of care organization already during therapy. The results should be used in each institution to deepen the knowledge of patients' symptom burden and needs, and thus to improve treatment options with regard to symptoms, but also patient care, and to respond appropriately to patients' needs.

## Data Availability

Data are only available upon request.
